# The future of animal protein: feeding a hungry world

**DOI:** 10.1093/af/vfaa033

**Published:** 2020-10-30

**Authors:** Scott J Eilert

**Affiliations:** Cargill Protein North America, Wichita, KS

The task is simple…we must feed approximately 10 billion people on this planet by the year 2050. We must do so with the same basic level of natural resources that are currently available for use today. As a part of this challenge, we must recognize that as the economies of developing nations mature and the wealth of these nations increases, so does the consumption of animal protein. As it pertains to animal protein, we must be able to do this under mounting pressures of labor, arable land, and water scarcity. Additionally, we must do so under increased regulatory and societal scrutiny of the very technologies that have enabled past advancements in resource utilization, productivity, and environmental stewardship. Finally, we must be able to keep up with the growing populations and the wealth of nations while making sure that we continually protect the welfare of the animals that will ultimately make up our diets.

To paraphrase an often-used quote, this task may be simple, but it is not easy. As myself and Dr. Anna Dilger were preparing for this issue (Figure 1), we spoke about the theme of the articles that we wished to feature. In those discussions, we quickly settled on the idea of assembling contributions that would address the theme of the challenges and opportunities faced by the animal protein industry as we meet the obligation of feeding 10 billion people by 2050.

**Figure 1.**
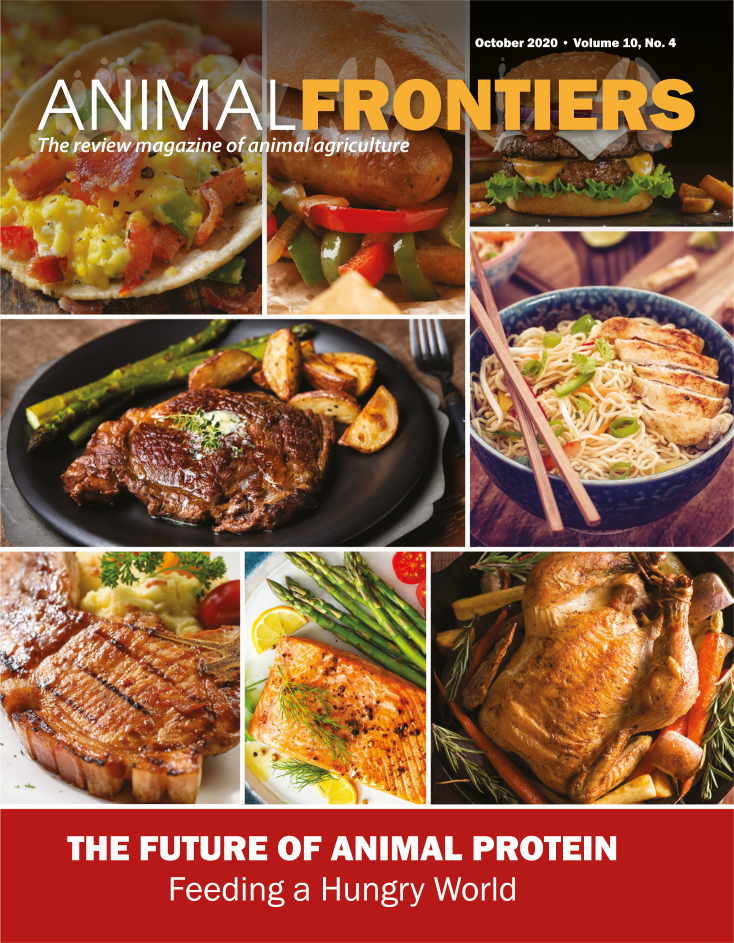
Cover image of october 2020 *Animal Frontiers*.

There are numerous environmental challenges that must be addressed as animal protein continues to be a part of feeding our global population. This issue includes two contributions that address the topic of greenhouse gas emissions of commercial livestock production. Dr. Judith Capper (Capper, 2020) explores the topic of the advancements that have been made in livestock productivity, which have also enabled lower greenhouse gas emissions from these production systems. Her paper also addresses the battle between the true science of sustainability versus the popular societal pressures against technologies and production systems that enable environmental impact reductions. In a contribution from Australia, Davison et al. (2020) outline the potential of several technologies to further reduce methane reductions from ruminants. These authors discuss both the scientific principles and the economic feasibility of achieving a carbon-neutral status from Australian ruminant production by the year 2030.

As mentioned above, history has shown that as the wealth of citizens in a nation advances, so does the proportion of animal protein in their diets. Dr. Isabel Baltenweck and her colleagues from ILRI and ICARDA reviewed the growing economies of Asia and Africa and the role that animal protein is likely to play in these nations (Baltenweck et al., 2020). This paper details tangible steps to move towards sustainable livestock production that benefits economic growth.

Recently, the animal protein industry has been challenged by an outbreak of African Swine Fever in Asia. This outbreak serves a reminder of the importance of biosecurity and disease control strategies. In the paper by Woonwong et al. (2020), the impact of this outbreak in some of the leading swine producing countries in Asia is discussed. They address how this animal disease can transform an industry and the eating habits of an entire region. While these outbreaks are regional in nature, their supply chain impacts are felt globally. This topic is not limited to the continent of Asia, and the authors share their thoughts on the best preventative strategies to be deployed in the future.

Feeding this world requires technological advancement in the production of animals and the conversion of these animals to energy and protein for human diets. Dr. Shai Barbut (Barbut, 2020) reviews the evolution of meat processing (primary and secondary processing) and the impact of automation on this industry. His paper also focuses on the productivity advancements in these industries, and how much more advancement can take place with the use of advanced data integration from primary production through to distribution to the consumer. Advancements in the efficiency of production through better technology will be for naught, however, unless the issue of food waste is solved. Lipinski (2020) reviews the staggering quantities of food of all origins that are lost or wasted during production and ultimate consumption. His paper outlines the challenges of food loss and waste in developed and developing geographies. He focuses on the particular challenges of animal products. While meat, milk, fish, and eggs are among some of the lowest volume of food that is wasted on the globe, the impact of these losses are much greater due to their cost and level of inputs. He also outlines what companies and nations are doing to make sure that the fruits of the food system labors are ultimately realized for human benefit.

In this issue that looks to the future of the animal protein industry, we felt that it was important to also focus on the potential for animal protein production that does not rely upon the growth of an entire animal to create the resultant protein and energy for humans. Dr. Rhonda Miller (2020) outlines the emerging technology of cell-cultured meat, poultry, and fish. Not only does she outline the science and challenges of this technology, but she also discusses the state of this technology with a variety of companies and stakeholders in this field. We also felt that it was critical in this issue to explore the replacement of animal protein with vegetable-based alternatives. Ismail et al. (2020) outline some of the current and emerging technology that is being deployed to substitute or replace animal protein in traditionally processed meat products.

In closing, Dr. Anna Dilger and myself would like to thank all of the authors for their contributions to this important issue and the reviewers who assisted in their publications. We hope that you find this issue to be informative and instructive. We know that all of you reading this paper are committed to playing a role in addressing this simple, but not easy challenge…feeding a growing populations with a finite level of resources.

## About the Author

Scott Eilert currently serves as the Vice President of Customer Quality for Cargill Protein, North America. He has been with Cargill for the last 25 yr. His first 17 yr were spent in various Research and Development roles. For the last 8 yr, he held various leadership roles in Food Safety, Quality, and Regulatory. He received his MS and PhD from the University of Nebraska-Lincoln, specializing in Meat Science, and he holds a BS in Animal Science from Kansas State University. Scott has been active in numerous scientific and trade associations throughout his career.
